# Antibacterial and anti-virulence potential of plant phenolic compounds against *Vibrio parahaemolyticus*

**DOI:** 10.12688/f1000research.141268.1

**Published:** 2023-10-02

**Authors:** F. Javier Vazquez-Armenta, M. Olivia Aros-Corrales, M. Lizeth Alvarez-Ainza, A. Thalia Bernal-Mercado, J. Fernando Ayala-Zavala, Adrian Ochoa-Leyva, A. Alexis Lopez-Zavala

**Affiliations:** 1 Departamento de Ciencias Quimico Biologicas, Universidad de Sonora, Hermosillo, Sonora, 83000, Mexico; 2 Departamento de Investigacion y Posgrado en Alimentos, Universidad de Sonora, Hermosillo, Sonora, 83000, Mexico; 3 Coordinacion de Tecnologia de Alimentos de Origen Vegetal, Centro de Investigacion en Alimentacion y Desarrollo AC, Hermosillo, Sonora, 83304, Mexico; 4 Departamento de Microbiologia Molecular, Instituto de Biotecnologia, Universidad Nacional Autonoma de Mexico, Mexico City, Mexico City, 62210, Mexico

**Keywords:** anti-virulence, natural compounds, vibriosis, food safety

## Abstract

**Background:** *Vibrio parahaemolyticus* is a pathogenic bacterium that affects shrimp aquaculture; its infection can lead to severe production losses of up to 90%. On the other hand, plant phenolic compounds have emerged as a promising alternative to combat bacterial infections. The antibacterial and anti-virulence activity of the plant phenolic compounds quercetin, morin, vanillic acid, and protocatechuic acid against two strains of *V. parahaemolyticus* (Vp124 and Vp320) was evaluated.

**Methods:** The broth microdilution test was carried out to determine phenolic compounds' antibacterial activity. Moreover, the biofilm-forming ability of *V. parahaemolyticus* strains in the presence of phenolic compounds was determined by total biomass staining assay using the cationic dye crystal violet. The semisolid agar displacement technique was used to observe the effect of phenolic compounds on the swimming-like motility of *V. parahaemolyticus.*

**Results:** Results showed that phenolic compounds inhibited both strains effectively, with minimum inhibitory concentrations (MICs) ranging from 0.8 to 35.03 mM. Furthermore, at 0.125 – 0.5 × MIC of phenolic compounds, *V. parahaemolyticus* biofilms biomass was reduced by 63.22 – 92.68%. Also, quercetin and morin inhibited the motility of both strains by 15.86 – 23.64% (Vp124) and 24.28 – 40.71% (Vp320).

**Conclusions:** The results suggest that quercetin, morin, vanillic, and protocatechuic acids may be potential agents for controlling *V. parahaemolyticus.*

## Introduction

According to the Food and Agriculture Organization of the United Nations (FAO), shrimp farming represents 53% of world aquaculture production. ^1^ In recent years, stocking densities have increased, which has led to the proliferation of diseases and the appearance of new pathologies that directly affect the profitability of shrimp production systems. Twenty percent of the diseases that appear during shrimp farming are related to bacteria, including the genus *Vibrio.* ^2^ *Vibrio parahaemolyticus* stands out as the causal agent of Acute Hepatopancreatic Necrosis Disease (AHPND), initially defined as Early Mortality Syndrome (EMS). ^3^ This disease has been responsible for losses of about 90% of production and, in some cases, up to 100%, threatening seafood safety and human health. ^3^^,^^4^ The increasing prevalence of antibiotic-resistant strains has made the control of *V. parahaemolyticus* infections progressively challenging.

Once effective in controlling these infections, traditional antibiotics are now facing limitations due to the emergence of resistant strains and the negative environmental impacts resulting from their extensive use in aquaculture. ^5^ Moreover, the complex and dynamic nature of aquatic environments makes preventing the spread of bacterial infections in these systems difficult. Waterborne pathogens, such as *V. parahaemolyticus*, can quickly disseminate throughout aquaculture facilities, contaminating water sources and other organisms.

In addition, the biofilm-forming capacity of *V. parahaemolyticus* further complicates the control measures, as biofilms provide protection against antimicrobial agents and enable the bacteria to persist in the environment. ^6^ Biofilms are communities of microorganisms attached to biotic or abiotic surfaces embedded in a self-produced matrix consisting of polysaccharides, proteins, and nucleic acids. ^7^ In the inner part of the biofilms, the oxygen concentration is lower. Hence, an anaerobic atmosphere with a low pH develops, which causes a decrease in the activity of some antibiotics. ^8^ Other phenomena caused by these altered microenvironment conditions are decreased bacterial metabolism and dropped replication times. ^9^ Therefore, once the biofilm is formed, its eradication becomes complicated. This challenge requires the development of novel strategies and compounds capable of effectively inhibiting bacterial growth, biofilm formation, and motility, without causing significant environmental harm or promoting antibiotic resistance.

The use of natural compounds, such as phenolic compounds, offers a promising alternative due to their antimicrobial properties and potential to address the limitations of traditional antibiotics. ^10^ Phenolic compounds, which are secondary metabolites produced by a wide variety of plants, have shown antibacterial activity against Gram-negative ( *Escherichia coli*, *Salmonella* spp., *Vibrio* spp.) and Gram-positive ( *Listeria monocytogenes* and *Staphylococcus aureus*) pathogenic bacteria. ^11^^–^^13^ They are widely distributed in plants as defense mechanisms against microbial pathogens. Owing to their structural diversity and biological activity, phenolic compounds have gained increasing interest as potential antibacterial agents in various applications, including food preservation, agriculture, and human health. ^14^^,^^15^ For example, the flavonoids quercetin and myricetin showed a minimum inhibitory concentration (MIC) against *V. parahaemolyticus* of 0.125 and 0.25 mg/mL, respectively. ^16^ In comparison, catechin and isorhamnetin showed MIC values of 0.05 and 0.025 mg/mL, respectively, against *V. cholerae.* ^17^ Moreover, some of them, such as quercetin, catechin, ferulic acid, vanillic acid, and protocatechuic acid, have been shown to disrupt biofilm formation of pathogenic bacteria through different mechanisms of action, including inhibition of motility and EPS synthesis or/and disruption of intercellular communication. ^18^^–^^21^ However, the effect of these phenolic compounds on *V. parahaemolyticus* virulence factors, such as motility and biofilm formation capacity is unknown. Therefore, the present study aimed to evaluate (quercetin, morin, vanillic acid, and protocatechuic acid) phenolic compounds’ antibacterial and anti-virulence activity against a *V. parahaemolyticus* reference strain (CAIM 320; Vp320), and a strain isolated from white shrimp samples (Vp124). It is hypothesized that the selected phenolic compounds will significantly inhibit the growth, biofilm formation, and motility of *V. parahaemolyticus* strains, thus highlighting their potential as alternative agents for controlling infections and the persistence of this pathogen. Furthermore, the flavonoids (quercetin and morin) are expected to exhibit different mechanisms of action compared to the phenolic acids (vanillic and protocatechuic acids), which may offer unique advantages in tackling *V. parahaemolyticus.*

## Methods

### Strains and culture conditions

The human pathogenic reference strain CAIM 320 (Vp320) harboring the virulence genes *tlh* (thermolabile haemolysin), *tdh* (thermostable direct haemolysin), *trh* (TDH-related haemolysin), T3SS1: VP1680 (type III secretion system 1) and VPI and a strain isolated from shrimp, designated as Vp124 ^22^ were donated by the Laboratory of Clinical Microbiology of the University of Sonora. The studied strains were seeded on Thiosulfate Citrate Bile Sucrose agar (TCBS), reseeded in Trypticase Soy Broth (TSB) with 3% NaCl, and incubated at 37°C during the assays.

### Phenolic compounds

Standards (purity >95%) of gallic acid (G7384), vanillic acid (94770), protocatechuic acid (03930590), rutin (R5143), morin (M4008), and quercetin (Q4951) were purchased from Sigma-Aldrich.

### Antibacterial activity of phenolic compounds against *Vibrio parahaemolyticus*

To determine the antibacterial capacity of phenolic compounds against *V. parahaemolyticus*, an inoculum of each strain (Vp124 and Vp320) was prepared from exponential phase cultures (16 h in TSB + NaCl 3% at 37 °C), adjusting the optical density (600 nm) to 0.1 absorbance units, which is equivalent to 1 × 10 ^8^ CFU/mL. Concentrations of 0.8 mM - 40 mM of each compound (quercetin, morin, vanillic acid, protocatechuic acid, gallic acid, and rutin) were prepared in TSB + NaCl 3% at 37°C from dimethyl sulfoxide (DMSO) stocks. Subsequently, 5 μL of the adjusted inoculum and 295 μL of each concentration of the compounds were taken and placed in microplate wells (Costar 96) and incubated for 24 h at 37°C. The positive control was the same bacterial inoculum in TSB + NaCl 3% + DMSO 5%. As negative controls, each concentration of the tested compound and TSB + NaCl were placed in microplate wells without bacterial inoculum. The lowest compound concentration at which no visible growth was observed was considered the minimum inhibitory concentration (MIC). In addition, each compound's minimum bactericidal concentration (MBC) was determined by placing 50 μL aliquots from wells with three concentrations above the MIC on Mueller-Hinton agar + NaCl 3% plates. The MBC was considered the minimum concentration of each compound where no growth of *V. parahaemolyticus* was observed on the plate. ^23^

### Effect of phenolic compounds on *V. parahaemolyticus* biofilm formation

The biofilm-forming ability of *V. parahaemolyticus* strains in the presence of phenolic compounds was determined by total biomass staining assay using the cationic dye crystal violet. ^24^ An inoculum of each strain at an optical density of 0.1 units at 600 nm was prepared from exponential phase cultures. Each compound was dissolved in DMSO (5% of final volume) and added to TSB at 0.125, 0.25, and 0.5 × MIC. Subsequently, 5 μL of the inoculum and 295 μL of each concentration were taken, placed in microplate wells, and incubated for 24 h at 37 °C.

At the end of the incubation, the culture medium was removed from the microplate wells by aspiration. Three consecutive washes were performed with distilled water to remove weakly adherent cells, and then the microplate was allowed to dry at 50°C. After that, 300 μL of a 0.1% crystal violet (Sigma-Aldrich) solution was added to each well and allowed to stand for 45 min to stain the biomass of the formed biofilms. The crystal violet solution was then carefully removed, and three consecutive washes were carried out to remove the excess dye with distilled water, followed by drying at 50°C. Finally, 300 μL of 20% acetic acid was added to each well to solubilize the crystal violet for 15 min, and the absorbance of the solution was read at 594 nm. The absorbance of the solubilized violet crystal is proportional to the total biomass of the biofilms formed in each well. Each experiment was performed in triplicate, and the results were expressed as a percentage of the reduction of total biomass compared to the positive control (biofilms formed without the presence of phenolic compounds) according to the following equation:

$$\text{Biomass reduction}\;\left(\%\right)=\frac{\mathrm{Abs}\;{\text{positive control}}_{594}-\mathrm{Abs}\;{\text{treatment}}_{594}\;}{\mathrm{Abs}\;{\text{positive control}}_{594}}\times 100$$

### Effect of phenolic compounds on swimming motility of *V. parahaemolyticus*

The semisolid agar displacement technique was used to observe the effect of phenolic compounds on the swimming-like motility of *V. parahaemolyticus.* ^24^ Phenolic compounds were added to TSB + NaCl 3% medium at a final concentration of 0.5 × MIC. An inoculum of each *V. parahaemolyticus* strain from exponential phase cultures (18 h at 37°C in TSB + NaCl 3%) was prepared at a final 1 × 10 ^8^ CFU/mL concentration. Subsequently, 10 μL of bacterial suspension were taken and placed in the center of a Petri dish with semi-solid TSB + NaCl 3% agar (0.42% agar) and incubated for 16 h at 37°C. Finally, the diameter of motility halos was measured. The experiment was performed in triplicate, and cultures of each *V. parahaemolyticus* strain on TSB + NaCl 3% without phenolic compounds were used as positive controls.

### Statistical analysis

A complete randomized experimental design was used for all experiments. In the biofilm formation assay, the factors were the type of compound (quercetin, morin, vanillic acid, and protocatechuic acid) and the tested concentration (0.125, 0.25, and 0.5 × MIC), and the response variable was biomass reduction (%). In the motility assay, the factors were also the type of compound and concentration, and the response variable was motility inhibition (%). An analysis of variance (ANOVA) was performed, and where differences were found, multiple comparisons of means were performed by the Tukey-Kramer method at 95% confidence using the statistical software NCSS 2007.

## Results

### Antibacterial activity of phenolic compounds against *V. parahaemolyticus*

The MICs of the assessed compounds are shown in Table 1, where four of the six compounds were able to inhibit the growth of both *Vibrio* strains. The flavonoid quercetin was the most effective, presenting the lowest MIC (0.8 mM for both strains). The flavonoid morin and protocatechuic acid showed the same effect on both *Vibrio* strains, with MICs of 1.6 and 28.43 mM, respectively. On the other hand, vanillic acid presented a MIC equal to 32.73 mM for the Vp124 strain and 35.03 for the Vp320 strain. In contrast, rutin and gallic acid did not inhibit the growth of *V. parahaemolyticus* at the evaluated concentrations, 0 - 4 mM and 0-41.12 mM, respectively. For this reason, they were discarded in the following determinations. The minimum bactericidal concentration was also evaluated; however, this effect was not observed at any tested concentrations. The observed MICs of quercetin, morin, protocatechuic acid, and vanillic acid against both *Vibrio* strains support our hypothesis that certain phenolic compounds can effectively inhibit the growth of *V. parahaemolyticus.*

**Table 1.** Minimum inhibitory concentrations (MIC) and minimum bactericidal concentrations (MBC) of phenolic compounds against *V. parahaemolyticus.*

Phenolic compound	Strain			
	Vp124		Vp320	
	MIC (mM)	MBC (mM)	MIC (mM)	MBC (mM)
Quercetin	0.8	>1.6	0.8	>1.6
Morin	1.6	>2.4	1.6	>2.4
Rutin	>4.0	>4.0	>4.0	>4.0
Protocatechuic acid	28.43	>35.03	28.43	>35.03
Vanillic acid	31.73	>38.33	35.03	>41.12
Gallic acid	>41.12	>41.12	>41.12	>41.12

### Effect of phenolic compounds on *V. parahaemolyticus* biofilm formation

**Figure 1.**
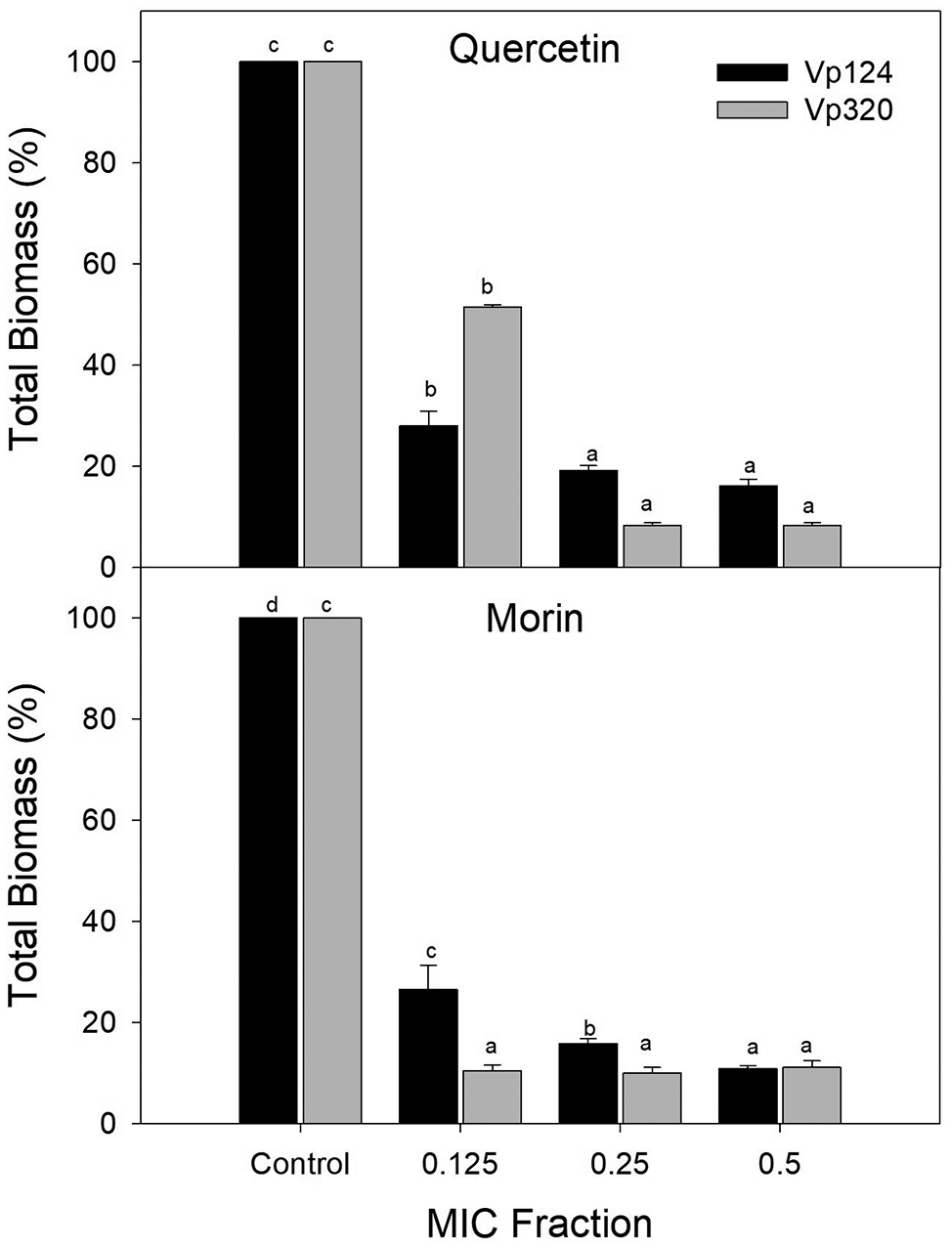
Effect of the flavonoids quercetin and morin on the total biomass of *V. parahaemolyticus* biofilms. Values represent mean ± standard error (n = 3). Different literals represent differences (p<0.05) between the evaluated concentrations for each strain.

To determine the effect of phenolic compounds on *V. parahaemolyticus* biofilm formation, sub-inhibitory concentrations of each compound were selected based on the MIC (0.125, 0.25, and 0.5 × MIC). Figure 1 shows the percentage of total biomass of *V. parahaemolyticus* biofilms formed at different concentrations of quercetin and morin. Both compounds were able to interfere with the biofilm formation process. For quercetin at 0.125 × MIC, a 71.97% reduction (p<0.05) was observed on the Vp124 strain, while no significant differences were found between 0.25 and 0.5 × MIC, achieving a reduction of 77.97-80.85% (p<0.05) respect to control. A similar pattern was observed in strain Vp320, where quercetin at 0.125 × MIC reduced 48.44% of the total biomass of *V. parahaemolyticus* biofilms, and for 0.25-0.5 × MIC, total biomass reduction was 85.31 – 91.66%. Regarding the effectiveness of morin, a reduction (p<0.05) in the total biomass of biofilms of strain Vp124 of 73.39%, 84.08%, and 89.07% was observed at concentrations of 0.125, 0.25, and 0.5 × MIC, respectively. While in the Vp320 strain, no differences were observed in the percentages of reduction at the evaluated concentrations (p>0.05), showing a reduction of 88.84-89.93 % compared to the control. The significant decrease of *V. parahaemolyticus* biofilm formation observed at sub-inhibitory concentrations of quercetin and morin supports our hypothesis that certain phenolic compounds can effectively disrupt biofilm formation processes.

**Figure 2.**
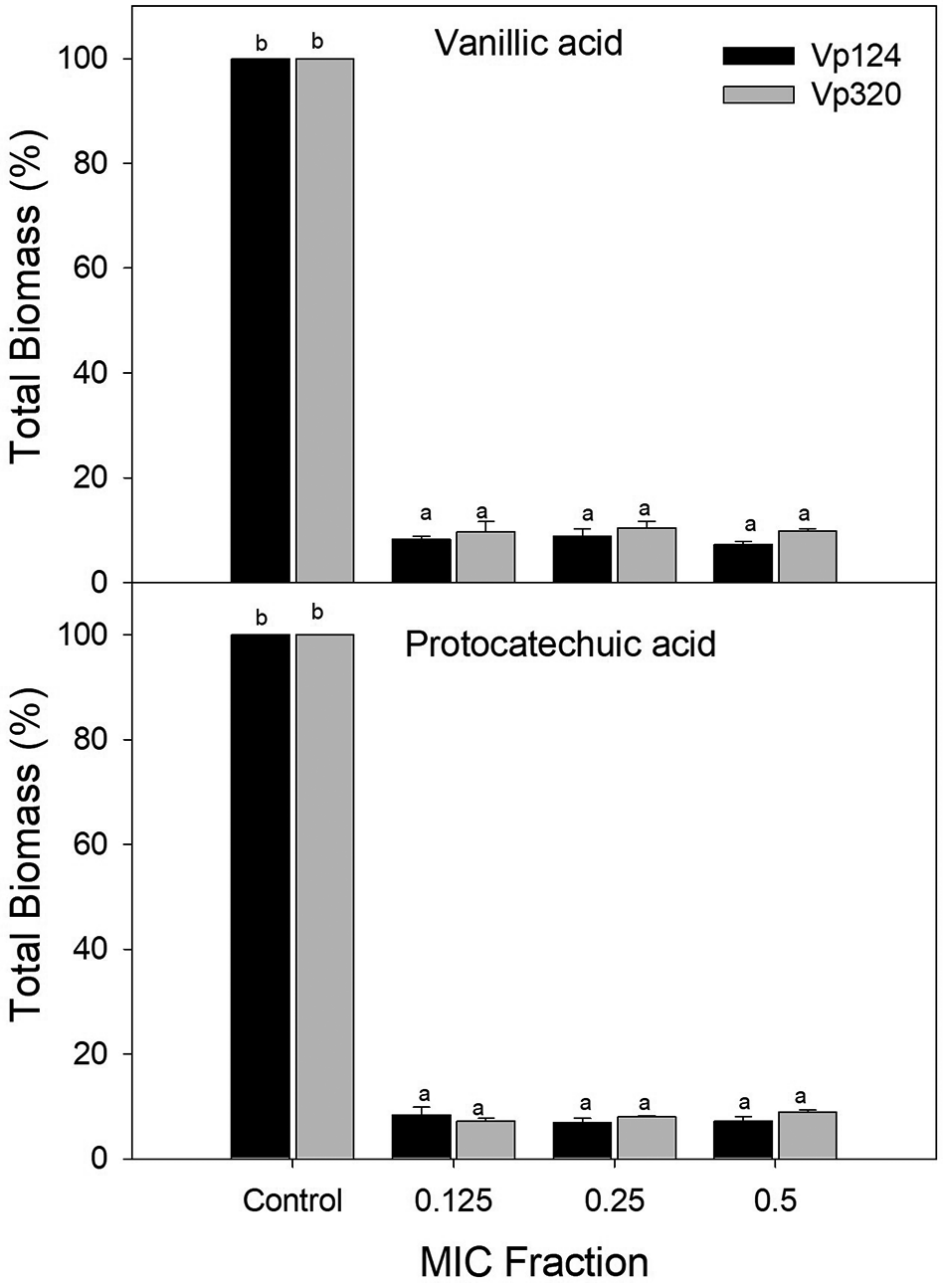
Effect of vanillic and protocatechuic acids on the total biomass of *V. parahaemolyticus* biofilms. Values represent mean ± standard error (n = 3). Different literals represent differences (p<0.05) between the concentrations evaluated for each strain **.**

Figure 2 shows the total biomass of biofilms formed by *V. parahaemolyticus* in the presence of vanillic and protocatechuic acids. Both phenolic acids inhibited the biofilm formation capacity of both strains compared to the control. However, no differences were found (p>0.05) between the evaluated concentrations. Vanillic acid reduced the total biomass of Vp124 biofilms by 85.92% to 92.68% and 89.55% - 90.27%for Vp320 strain. In comparison, protocatechuic acid inhibited 91.61% - 93.12% and 91.04% - 92.82% of the biofilm formation of Vp124 and Vp320 strains, respectively.

### Effect of phenolic compounds on the motility of *V. parahaemolyticus*

Table 2 shows the effect of quercetin, morin, vanillic and protocatechuic acids on the motility of Vp124 and Vp320 strains. At the tested concentrations, the flavonoids quercetin and morin inhibited the motility of both strains on soft agar (0.42% agar). In this assay, differences (p<0.05) were observed in the inhibition percentages of each strain. For the Vp124 strain, quercetin inhibited 15.86% of the displacement of the bacteria on soft agar; while an inhibition of 23.63% was observed for the Vp320 strain. In the presence of morin, a similar trend was observed, where Vp320 showed the highest (p<0.05) percentage of motility inhibition (40.71%), while for the Vp124 strain, the percentage of inhibition was 24.28%. However, phenolic acids at the evaluated concentration did not show the capacity to interfere with the motility of *V. parahaemolyticus* strains (p>0.05). These results demonstrated that the flavonoids quercetin and morin effectively inhibited the motility of *V. parahaemolyticus* strains Vp124 and Vp320, following the hypothesis that certain phenolic compounds can impact bacterial motility. On the other hand, phenolic acids, such as vanillic and protocatechuic acids, did not exhibit the same inhibitory effect on motility, highlighting the varying impacts of different phenolic compounds on *V. parahaemolyticus* motility.

**Table 2.** Effect of phenolic compounds at 0.5 × MIC on the *swimming* motility of *V. parahaemolyticus.* Values are mean ± standard error (n = 3).

Compound	Motility inhibition (%)	
	Vp124	Vp320
Quercetin	15.86 ± 0.87	23.63 ± 4.13
Morin	24.28 ± 1.02	40.71 ± 3.53
Vanillic	0	0
Protocatechuic acid	0	0

## Discussion

Phenolic compounds are known for their antimicrobial activity against various pathogenic bacteria, including Gram-negative and Gram-positive species. ^10^^,^^11^ In this study, vanillic and protocatechuic acids, along with the flavonoids quercetin and morin, effectively inhibited the growth of *V. parahaemolyticus.* Notably, flavonoids exhibited 20-40 times stronger antibacterial activity than phenolic acids, as evidenced by their lower MICs, which aligns with findings from previous research. Abuga *et al*. ^16^ demonstrated that quercetin and myricetin inhibited *V. parahaemolyticus*, with MICs of 0.4 and 0.8 mM, respectively. Similarly, Tinh *et al*. ^12^ evaluated the antibacterial activity of phenolic compounds against 96 *V. parahaemolyticus* isolates from Pacific white shrimp ( *L. vannamei*) in Thailand. Among the tested compounds, vanillic acid displayed significant bactericidal activity, with MICs and MBCs ranging from 6.09 to 12.18 mM. Pyrogallol, however, exhibited the strongest antibacterial properties, with MICs and MBCs between 0.25 and 2.02 mM. Wu *et al*. ^25^ also investigated the MIC of 3- *p*- *trans*-coumaroyl-2-hydroxyquinic acid against various pathogenic bacteria, including *V. parahaemolyticus.* Their results showed MICs ranging from 7.06 to 28.24 mM for all evaluated pathogenic bacteria, with a MIC of 14.12 mM for *V. parahaemolyticus.* In conjunction with our current research findings, the outcomes of these earlier studies underscore the potential of phenolic compounds as effective antibacterial agents against *V. parahaemolyticus.* Our study contributes to the growing body of evidence by demonstrating the comparative effectiveness of flavonoids and phenolic acids, highlighting the promising role of these natural compounds in combating *V. parahaemolyticus* infections.

One notable aspect of these results is the direct comparison between flavonoids and phenolic acids regarding their antibacterial activity against *V. parahaemolyticus.* While previous research has separately investigated various phenolic compounds for their antimicrobial properties, our study offers a unique contribution by evaluating and contrasting the effectiveness of both flavonoids and phenolic acids. The results of our study emphasize that flavonoids, such as quercetin and morin, demonstrated 20-40 times stronger antibacterial activity than phenolic acids, as evidenced by their lower MICs. This novel finding highlights the potential advantages of using flavonoids over phenolic acids in developing new strategies to control and prevent *V. parahaemolyticus* infections in aquaculture systems. Additionally, our study contributes to the growing body of evidence supporting using natural compounds as alternatives to traditional antibiotics, which is particularly important in increasing antibiotic resistance.

The antibacterial activity of phenolic compounds is closely related to their physicochemical properties and the ability to interact with the lipid membrane and intracellular targets. Phenolic acids can alter the morphology of treated cells and cause hyperpolarization of the lipid membrane with its consequent loss of integrity. ^25^^,^^26^ While flavonoids can intercalate in different regions of lipid bilayers, causing changes in their fluidity, this effect depends on the number and position of hydroxyl groups. ^27^ Wu *et al*. ^28^ found a strong and positive correlation ( *r* = 0.921) between the antimicrobial activity of flavonoids against *E. coli* and their ability to reduce the fluidity of lipid membranes. Furthermore, flavonoids can inhibit essential enzymes for bacterial growth, *e.g.*, quercetin, kaempferol, luteonin, galangin, and myricetin are inhibitors of DNA gyrase (IC _50_ = 0.037-1.18 mg/mL). ^29^ Also, quercetin and apigenin can inhibit d-alanine:d-alanine ligase, the enzyme responsible for the binding of alanine residues during the assembly of peptidoglycan precursors. ^30^

Biofilms enable pathogenic bacteria to survive in adverse environments, such as nutrient scarcity and exposure to antimicrobial agents. ^7^^,^^31^ *V. parahaemolyticus* can form biofilms on various surfaces in aquaculture-related settings, acting as a reservoir that compromises the safety of aquaculture products. ^6^ Our study reveals that vanillic and protocatechuic acids and the flavonoids quercetin and morin can effectively interfere with *V. parahaemolyticus* biofilm formation ( Figures 1 and 2), contributing new insights into the potential of these compounds. Liu *et al*. ^21^ demonstrated that vanillic acid reduced the total biomass of *V. alginolyticus* biofilms by approximately 65% at a concentration of 2.9 mM. Additionally, 2,6-di-tert-butyl-4-methylphenol, found in garlic, green algae, and cyanobacteria, inhibited the biofilm formation of *V. harveyi, V. parahaemolyticus*, and *V. vulnificus* by 80%, 83%, and 80%, respectively. ^32^ Moreover, ethanolic extracts of ginger and its major compounds, 6-gingerol, 8-gingerol, and 6-shogaol, effectively inhibited *V. parahaemolyticus* biofilm formation, with 6-shogaol exhibiting the highest efficacy by reducing biofilm biomass by up to 80% at a concentration of 0.15 mM. ^33^ This study breaks new ground by assessing the anti-biofilm activity of selected flavonoids and phenolic acids against *V. parahaemolyticus*, demonstrating their potential as anti-biofilm agents in combating infections in aquaculture systems. This research significantly expands on prior findings, offering a deeper and more extensive understanding of phenolic compounds' capabilities in mitigating biofilm-associated risks within the aquaculture industry.

Motility is a key virulence factor in the *Vibrio* genus, essential in the initial adhesion to abiotic surfaces and subsequent biofilm formation. ^34^^,^^35^ Our study reveals that flavonoids quercetin and morin inhibited *V. parahaemolyticus* swimming motility, while vanillic and protocatechuic acids did not exert any inhibitory effects ( Table 2). *V. parahaemolyticus* exhibits swimming motility in aquatic environments, driven by a single polar flagellum, ^36^ which relies on the expression of approximately 60 genes organized in at least 11 operons. ^37^^,^^38^ Roy *et al*. ^39^ reported that quercetin (0.09-0.36 mM) repressed *flaA* and *flgL* genes encoding flagellin, inhibiting *V. parahaemolyticus* motility and biofilm formation on shrimp and crab tissues. Our results corroborated these findings and further suggested that quercetin and morin may impair biofilm formation due to their motility-inhibiting effects. Notably, Vp124 (shrimp isolate) and Vp320 (clinical isolate) exhibited differential responses to flavonoids, with Vp124 being more resistant. Previous studies have indicated that mutations in sodium-type flagellar motor genes ( *motX* and *motY*) confer resistance to known bacterial motility inhibitors, such as phenamil and amiloride. ^40^ Investigating a potential similar resistance mechanism in these strains could be worthwhile. In contrast, the lack of inhibitory effects of vanillic and protocatechuic acids on *V. parahaemolyticus* motility suggests that they may interfere with biofilm formation through alternative mechanisms, such as modifying the physicochemical properties of the bacterial surface. ^18^^,^^20^ This study provides insights into the potential role of phenolic compounds as motility inhibitors and enhances our understanding of biofilm disruption strategies in the aquaculture industry.

The different responses observed with flavonoid treatments between the Vp124 strain (isolated from shrimp), and the reference strain (clinical isolate) warrant further investigation to elucidate the underlying resistance mechanisms. It is plausible that genetic or phenotypic differences between the two strains may contribute to their varying susceptibilities to the tested compounds. The disparity in response could also be attributed to differences in gene expression profiles, metabolic pathways, or the presence of specific efflux pumps that confer resistance to these flavonoids. ^41^ Further research employing genomic, transcriptomic, and proteomic approaches could help to identify the key genetic determinants and molecular pathways responsible for these differential responses. By understanding the resistance mechanisms in play, it may be possible to tailor more targeted and effective interventions for controlling *V. parahaemolyticus* infections in different settings, such as aquaculture and clinical environments. Despite the valuable insights gained from this study, some limitations should be acknowledged. Investigating a broader range of phenolic compounds and additional strains, including environmental isolates, would help to create a more complex panorama. In addition, the specific mechanisms exerted by phenolic compounds in motility and biofilm formation could go deeper into exploring the molecular targets and pathways involved in the observed inhibitory effects. Additionally, the potential synergistic or antagonistic interactions between the tested compounds and their combination with other antimicrobial agents should be investigated to identify more effective biofilm disruption and motility inhibition strategies. Future studies should incorporate *in vivo* and field experiments to evaluate the efficacy and feasibility of implementing these phenolic compounds. Addressing these limitations will deepen our understanding of the potential applications of phenolic compounds in controlling *V. parahaemolyticus* infections and mitigating biofilm-associated risks in the aquaculture industry.

## Conclusions

This study provides valuable insights into the potential of phenolic compounds, particularly quercetin and morin, in controlling *V. parahaemolyticus* infections by inhibiting biofilm formation and motility. Quercetin and morin exhibited strong antibacterial activity compared to vanillic and protocatechuic acids. Moreover, both flavonoids effectively reduced biofilm formation and motility, supporting the hypothesis that these compounds can interfere with key virulence factors in *V. parahaemolyticus.* Finally, further research should address the study's limitations and focus on the underlying molecular mechanisms, synergistic or antagonistic interactions between the tested compounds, and *in vivo* efficacy to fully harness the potential of these phenolic compounds in combating *V. parahaemolyticus* infections.

## Data Availability

Figshare: Underlying data supporting the results of the manuscript “Antimicrobial and Anti-Virulence Potential of Plant Phenolic Compounds Against Vibrio parahaemolyticus” https://doi.org/10.6084/m9.figshare.24123315.v4. ^42^ Data are available under the terms of the CC BY 4.0 Attribution International license (CC BY 4.0).
