# Supplementary material for: Antibacterial and anti-virulence potential of plant phenolic compounds against Vibrio parahaemolyticus
Source: F1000Res. 2024 Jul 3;12:1256. Originally published 2023 Oct 2. [Version 2] doi: 10.12688/f1000research.141268.2 (PMC11437291; doi:10.12688/f1000research.141268.2)
Supplement: Peer review history

# Reviewer report: Reviewer response for version 2

**DOI:** 10.5256/f1000research.168486.r316670

**Published:** 2024-09-27

**Authors:** Nguyen Ngoc Phuoc

**Affiliations:** 1 University of Agriculture and Forestry, Hue University, Phung Hung, Vietnam

This study investigates the antibacterial and anti-virulence effects of the plant phenolic compounds quercetin, morin, vanillic acid, and protocatechuic acid on two strains of *Vibrio parahaemolyticus* (Vp124 and Vp320). As a proof-of-concept, it has the potential to support the hypothesis that these compounds significantly inhibit growth, biofilm formation, and motility of V. parahaemolyticus strains. The findings could inform larger studies involving additional *V. parahaemolyticus *strains. The experiments are well-designed and persuasive, providing a promising method for assessing the antibacterial and anti-virulence potential of these compounds. Results are clearly presented, and the discussion effectively highlights how this study contributes new knowledge to the existing literature in the field. However, the handling of individual details in the article is not perfect. There are some minor suggestions, which are specified below:

1. The introduction highlights the importance of investigating the antibacterial and anti-virulence effects of plant phenolic compounds. While this study focuses on two virulence factors of *V. parahaemolyticus*-biofilm formation and motility-there is an emphasis on biofilm formation, with insufficient discussion on motility. It would strengthen the introduction to include information on why studying bacterial motility is essential, as it plays a crucial role in the pathogen's ability to invade host tissues and contribute to infection dynamics. Providing this context will enhance the rationale for the study's
2. The method section for the antibacterial activity of phenolic compounds should include the preparation method for each compound in DMSO. This will help readers understand why the positive control was performed in TSB + NaCl 3% + DMSO 5%. Additionally, please specify the percentage of NaCl in the negative control treatment in the sentence: “As the negative controls, each concentration of the tested compound and TSB + NaCl were placed in microplate wells without bacterial inoculum.”
3. The limitation of small sample size should be mentioned in the discussion section to provide a more comprehensive assessment of the study's findings.

Is the work clearly and accurately presented and does it cite the current literature?

Yes

If applicable, is the statistical analysis and its interpretation appropriate?

Yes

Are all the source data underlying the results available to ensure full reproducibility?

Yes

Is the study design appropriate and is the work technically sound?

Yes

Are the conclusions drawn adequately supported by the results?

Yes

Are sufficient details of methods and analysis provided to allow replication by others?

Yes

Reviewer Expertise:

My research area focuses on the pathology of aquatic animals, particularly their immunology. I study the application of immunostimulants to combat diseases in fish and shrimp, exploring how these compounds can enhance immune responses and improve overall health in aquaculture.

I confirm that I have read this submission and believe that I have an appropriate level of expertise to confirm that it is of an acceptable scientific standard.

# Reviewer report: Reviewer response for version 1

**DOI:** 10.5256/f1000research.154697.r260789

**Published:** 2024-05-21

**Authors:** Chumporn Soowannayan

**Affiliations:** 1 Center of Excellence for Shrimp Molecular Biology and Biotechnology (CENTEX SHRIMP), Faculty of Science, Mahidol University, Salaya, Nakhon Pathom, Thailand

The work is interesting and could be helpful to aquatic animal and human health research or bacteria disease research in general. The comments and questions are posted in the attached main manuscript file which can be viewed here.

Is the work clearly and accurately presented and does it cite the current literature?

Partly

If applicable, is the statistical analysis and its interpretation appropriate?

I cannot comment. A qualified statistician is required.

Are all the source data underlying the results available to ensure full reproducibility?

Yes

Is the study design appropriate and is the work technically sound?

Partly

Are the conclusions drawn adequately supported by the results?

Yes

Are sufficient details of methods and analysis provided to allow replication by others?

Partly

Reviewer Expertise:

Fish and shrimp diseases

I confirm that I have read this submission and believe that I have an appropriate level of expertise to confirm that it is of an acceptable scientific standard, however I have significant reservations, as outlined above.

# Response

**Published:** 2024-06-26

**Authors:** Francisco Javier Vázquez Armenta

**Affiliations:** DCQB, Universidad de Sonora, Hermosillo, Sonora, Mexico

Dear reviewer,

 We appreciate your valuable feedback, which has greatly enhanced the quality and clarity of our manuscript. Below, we address each of your major and minor concerns point-by-point.

**Please check if this sentence is correct. If I'm not wrong, the world cultured shrimp production in 2023 was around 6 million tons, but the total aquaculture production of that year was around 96 million tons.**

**Response:** We appreciate your observations. The sentence was modified to indicate that this percentage is relative to crustacean production. Also, the reference was updated to the last published report.

**Many Vibrio species can cause AHPND, not just V. parahaemolyticus.**

**Response: **We appreciate your observations and agree that many AHPNDs are caused by vibrio and non-vibrio species carrying the pVA-1 plasmid, which expresses the PirAB toxin.

 The paragraph was modified as follows:

*“Vibrio parahaemolyticus* is considered of veterinary importance in shrimp farming, as some strains can infect shrimp, causing various diseases such as vibriosis. Strains that have acquired the pVA-1 plasmid, encoding the PirAB toxin, are of special concern because they cause Acute Hepatopancreatic Necrosis Disease (AHPND), initially known as Early Mortality Syndrome (EMS) ^3^. This atypical vibriosis has been responsible for losses of about 90% of production and, in some cases, up to 100%.”

**Are the bacteria causing AHPND the same as those causing disease in humans? If I'm right, they are different.**

**Response: **The bacteria causing Acute Hepatopancreatic Necrosis Disease (AHPND) in shrimp are typically strains of Vibrio parahaemolyticus, which are different from the strains that usually cause disease in humans. The paragraph was restructured to mention only the consequences of V. parahaemolyticus infections in shrimp farming.

**What was the concentration of each compound in DMSO? What were the final concentrations of the DMSO left in the final cultures?**

**Response: **The concentration of the DMSO stocks varies based on the evaluated compound, and the final cultures (both treatments and controls) had a 5% DMSO concentration.

**How many replicates were performed for each compound and each concentration?**

**Response: **MIC and MBC were made in triplicate.

**Is this the correct reference?**

**Response: **The correct reference was included.

**How many replicates?**

**Response: **Each experiment was performed in triplicate.

**Why use standard error, not standard deviation?**

**Response:** We have chosen to display the standard error on the graphs to show the accuracy of the estimate of the population mean, thereby providing a clear indication of the precision of our results. Additionally, using standard error helps readers visualize the differences among treatments, facilitating an intuitive comparison of the variability and significance between different groups.

**How do we know if these two are the cause or the effect?**

**Response:** *Vibrio parahaemolyticus *has two types of motilities: swimming and swarming. The former uses a polar flagellum in liquid media, and the latter uses lateral flagella in solid media or viscous environments. In our study, we focused on swimming motility since this kind of movement is related to the initial attachment of Vibrio to solid surfaces to start biofilm development (Zhang et al. 2023, Lu et al. 2021).

 Thank you again for your comments.

 Sincerely,

 The authors

 References:

 Lu, Renfei, Junfang Sun, Yue Qiu, Miaomiao Zhang, Xingfan Xue, Xue Li, Wenhui Yang, Dongsheng Zhou, Lingfei Hu, and Yiquan Zhang. 2021. "The quorum sensing regulator OpaR is a repressor of polar flagellum genes in Vibrio parahaemolyticus." *Journal of Microbiology* 59 (7):651-657. doi: 10.1007/s12275-021-0629-3.

 Zhang, Yiquan, Tingting Zhang, Yue Qiu, Miaomiao Zhang, Xiuhui Lu, Wenhui Yang, Lingfei Hu, Dongsheng Zhou, Bo Gao, and Renfei Lu. 2023. "Transcriptomic Profiles of Vibrio parahaemolyticus During Biofilm Formation." *Current Microbiology* 80 (12):371. doi: 10.1007/s00284-023-03425-7.

# Reviewer report: Reviewer response for version 1

**DOI:** 10.5256/f1000research.154697.r272299

**Published:** 2024-05-15

**Authors:** Saharuetai Jeamsripong

**Affiliations:** 1 Department of Veterinary Public Health, Faculty of Veterinary Science, Chulalongkorn University, Bangkok, Bangkok, Thailand

**Antibacterial and anti-virulence potential of plant phenolic compounds against *Vibrio parahaemolyticus***

 This study investigated antibacterial and anti-virulence effects of the plant phenolic compounds quercetin, morin, vanillic acid, and protocatechuic acid on two strains of *V. parahaemolyticus* (Vp124 and Vp320).

 Major concerns

- No reference protocols and equations are provided in the materials and methods section.
- Details of media and reagents used, including company, city, and country should be included.
- Why did the authors choose to test only two strains of *V. parahaemolyticus*? Testing only two isolates of *V. parahaemolyticus* is a major limitation due to the small sample size, which may affect the accuracy and generalizability of the study. This limitation should be addressed.
- How many replicates were used in the experiments?
- The rationale for selecting phenolic compounds over other chemicals should be explained.
- The antibiotics and their concentrations used for treatment of *Vibrio *infection comparison with phenolic compounds should be specified. Additionally, the challenges of using plant phenolic compounds, including quantity of phenolic compounds and field application feasibility, should be discussed.

Minor concerns

- Please verify the unit of MIC in the abstract.
- There is an inconsistency in the results for vanillic acid in the table (31.73) and the text (32.73). This discrepancy needs to be corrected.
- Data regarding the antibacterial activity of quercetin and morin, which is 20-40 times higher than that of phenolic acids, is redundant in discussion and should be revised.

Is the work clearly and accurately presented and does it cite the current literature?

Partly

If applicable, is the statistical analysis and its interpretation appropriate?

Yes

Are all the source data underlying the results available to ensure full reproducibility?

Partly

Is the study design appropriate and is the work technically sound?

No

Are the conclusions drawn adequately supported by the results?

Yes

Are sufficient details of methods and analysis provided to allow replication by others?

Partly

Reviewer Expertise:

antimicrobial resistance, aquaculture, epidemiology, food safety

I confirm that I have read this submission and believe that I have an appropriate level of expertise to confirm that it is of an acceptable scientific standard, however I have significant reservations, as outlined above.

# Response

**Published:** 2024-06-26

**Authors:** Francisco Javier Vázquez Armenta

**Affiliations:** DCQB, Universidad de Sonora, Hermosillo, Sonora, Mexico

Dear reviewer,

 We want to express our gratitude for your constructive comments, which have significantly helped us improve the quality and clarity of our manuscript. Here, we provide a point-by-point response to major and minor concerns.

**Major concerns**

- **No reference protocols and equations are provided in the materials and methods section.**

**Response: **we appreciate your observations. The materials and methods section was improved, including the reference in the first sentence of each section.

- **Details of media and reagents used, including company, city, and country should be included.**

**Response: **Details of media and reagents were included in the materials and method section.

- **Why did the authors choose to test only two strains of *V. parahaemolyticus*? Testing only two isolates of *V. parahaemolyticus* is a major limitation due to the small sample size, which may affect the accuracy and generalizability of the study. This limitation should be addressed.**

**Response: **we appreciate your feedback and acknowledge the limitation regarding the sample size of *V. parahaemolyticus* strains tested in our study. We selected two strains with distinct phenotypic and genotypic profiles to provide a starting point for understanding the potential variability within this species. However, we agree that the small sample size limits the generalizability of our findings. We plan to expand our study to include a larger and more diverse set of *V. parahaemolyticus* isolates to address this. This will allow us to validate our preliminary results and enhance the accuracy and applicability of our conclusions. We will also explicitly mention this limitation in the discussion section of our manuscript and outline our future research plans to address this issue. By doing so, we aim to provide a more comprehensive understanding of *V. parahaemolyticus* and contribute to the broader body of knowledge in this field.

- **How many replicates were used in the experiments?**

**Response: **All experiments were done in triplicate.

**The rationale for selecting phenolic compounds over other chemicals should be explained.**

**Response: **thank you for your observation. We appreciate the opportunity to clarify our rationale for selecting phenolic compounds for this study.

 The selection of phenolic compounds was driven by the proven antimicrobial properties, safety profile, natural origin, and the potential for synergistic effects.  Phenolic compounds exert antimicrobial effects by disrupting cell membranes, inactivating essential enzymes, and interfering with microbial metabolism, enhancing their inhibitory potential against bacteria. Their natural occurrence in plants and presence in the human diet generally make them safer than synthetic chemicals, aligning with the trend toward plant-based antimicrobials. Previous research has demonstrated their efficacy against various *Vibrio* species, and the current study aims to confirm their effectiveness specifically against *V. parahaemolyticus*. Additionally, phenolic compounds can work synergistically with other antimicrobial agents, suggesting their potential in developing more effective treatment strategies.

 We will incorporate this rationale into the manuscript to provide a clearer understanding of our choice and strengthen the context of our research. Thank you again for your insightful feedback.

**The antibiotics and their concentrations used for treatment of *Vibrio *infection comparison with phenolic compounds should be specified. Additionally, the challenges of using plant phenolic compounds, including quantity of phenolic compounds and field application feasibility, should be discussed.**

**Response: **we appreciate the opportunity to address these important points and provide additional clarity in our study. We recognize the significance of discussing the concentration of antibiotics used in aquiculture and the challenges of using plant-derived phenolic compounds to treat Vibrio infections. Accordingly, we have included a detailed paragraph in the discussion section to address these issues comprehensively.

**Minor concerns**

- Please verify the unit of MIC in the abstract.

**Response: **the MIC unit in the abstract was verified.

- There is an inconsistency in the results for vanillic acid in the table (31.73) and the text (32.73). This discrepancy needs to be corrected.

**Response: **we appreciate your observation; the correct value of MIC is 31.73 mM.

- Data regarding the antibacterial activity of quercetin and morin, which is 20-40 times higher than that of phenolic acids, is redundant in discussion and should be revised.

**Response: **we appreciate your observation; this issue was addressed.

 Thank you again for your comments.

 Sincerely,

 The authors
